# Access to prevention of mother‐to‐child transmission of HIV along HIV services cascade through integrated active case management in 15 operational districts in Cambodia

**DOI:** 10.1002/jia2.25388

**Published:** 2019-10-20

**Authors:** Sovannarith Samreth, Vannak Keo, Romaing Tep, Angheng Ke, Vichea Ouk, Bora Ngauv, Sovatha Mam, Laurent Ferradini, Penh S Ly, Chhi V Mean, Therese Delvaux

**Affiliations:** ^1^ National Center for HIV/AIDS, Dermatology and STD Phnom Penh Cambodia; ^2^ University of Health Science Phnom Penh Cambodia; ^3^ FHI 360 Phnom Penh Cambodia; ^4^ Institute of Tropical Medicine Antwerp Belgium

**Keywords:** ARV, children, HIV prevention, vertical transmission, pediatric, ART, maternal, mother‐to‐child transmission, PMTCT

## Abstract

**Introduction:**

Following the introduction of option B+ in 2013, and with the perspective of eliminating mother‐to‐child transmission of HIV by 2025, Cambodia has implemented an integrated active case management (IACM) approach since 2014 to improve the notification and follow‐up of all HIV‐infected cases including pregnant women, and to ensure access to and use of the full prevention of mother‐to‐child transmission (PMTCT) service package by HIV‐infected pregnant women and their HIV‐exposed infants. This study aimed to analyse PMTCT cascade data in 15 operational districts (ODs) implementing the IACM approach in Cambodia.

**Methods:**

We analysed PMTCT cohort data from 15 ODs implementing IACM approach between 1 January 2014 and 31 December 2016. We measured key indicators along the PMTCT cascade and compared them to available (cross‐sectional) PMTCT indicators during the 2011 to 2013 period.

**Results:**

During the period 2014 to 2016, among 938 identified HIV‐infected pregnant women, 308 (32.8%) were tested HIV positive during their pregnancy, 9 (1.0%) during labour, while the remaining 621 (66.2%) were women on antiretroviral therapy (ART) who became pregnant. During the study period, 867 (92.4%) of the 938 women received ART during pregnancy and labour. Subsequently, 456 (85.6%) of the 533 HEI born and alive during the study period received 6‐week antiretroviral (ARV) prophylaxis, 390 (76.6%) and 396 (77.8%) of the 509 infants aged six weeks or older received cotrimoxazole prophylaxis and HIV‐DNA PCR test respectively. Among the 396 HEI who received HIV‐DNA PCR test, 7 (1.8%) were found HIV positive. The comparison with cross‐sectional PMTCT indicator obtained during the previous 2011 to 2013 period in the same 15 ODs, showed a significant increase in ARV uptake among HIV‐infected pregnant women (from 72.3% to 92.4%), in cotrimoxazole uptake (from 41.6% to 73.2%), and in HIV‐DNA PCR testing coverage among HEI (from 41.2% to 74.3%).

**Conclusions:**

The implementation of option B+ and IACM may have contributed to the improvement of the PMTCT cascade in Cambodia. However, some gaps in accessing PMTCT services along the HIV cascade persist and need to be addressed.

## 1 Introduction

Elimination of paediatric HIV infections and ensuring survival and long‐term wellbeing of HIV‐infected women are of high international public health concern 1, 2, 3, 4. Cambodia has made large progress in responding to the HIV epidemic 5. By the end of 2016, of the 70,721 total estimated number of people living with HIV, 58,338 (82.5%) had known their HIV status and were enrolled in care; among them 56,754 (97.3%) were on antiretroviral therapy (ART), and 45,064 (79.4%) had their HIV viral load suppressed 5, 6.

Remarkable progress has also been observed in maternal/reproductive and child health including prevention of mother‐to‐child transmission of HIV (PMTCT). The country's maternal mortality ratio declined from 472 per 100,000 live births in 2005 to 170 per 100,000 live births in 2014 7. Similarly, under‐5 child mortality rate decreased substantially from 124 deaths per 1000 live births in 2000 to 34 deaths per 1000 live births in 2014 7. In terms of utilization of services, in 2014, 95% of pregnant women attended at least one antenatal care (ANC) visit and 83% delivered at a heath facility 7. According to UNAIDS estimates, 79% of estimated HIV‐infected pregnant women received antiretroviral (ARV) regimens for PMTCT at the end of 2016 8.

To increase the coverage of PMTCT services, the Linked Response approach, a comprehensive intervention, which relied on the close cooperation between HIV and maternal/reproductive and child health services and on community mobilization, was implemented in 2008 9, 10. Building on the success of a pilot project implemented in five operational districts (ODs), the approach was scaled‐up countrywide in 2009 to 2011 10. At the same time, in 2010, Cambodia adopted the WHO PMTCT guidelines Option B that is triple ARV regimen or ART for all HIV‐infected pregnant women from 14 weeks of gestation for the duration of pregnancy and the breastfeeding period regardless of CD4 cell count 11.

Cambodia has committed to eliminating the transmission of HIV from mother to child by 2025 and has endorsed the elimination of mother‐to‐child transmission (EMTCT) strategy 12, 13. According to global WHO guidance on criteria for validation of EMTCT of HIV, the mother‐to‐child transmission (MTCT) rate of HIV has to be reduced to less than 5%, the coverage of pregnant women attending at least one ANC and pregnant women who know their HIV status have both to be maintained at levels above 95%, and the ARV coverage of HIV‐positive pregnant women has to be above 90% 12. The country's commitment to the EMTCT strategy led to introducing the option B+ for PMTCT which includes lifelong ART following a positive HIV test during pregnancy, childbirth or breastfeeding period, in April 2013 13. In addition, to strategically address remaining gaps and develop cost‐effective interventions in the context of decreasing external financial support, Cambodia also adopted an Integrated Active Case Management (IACM) approach in 2013. This approach supports the notification of new HIV cases, including pregnant women, and ensures that each mother‐baby pair receive the full package of PMTCT services, with accelerated enrolment in paediatric AIDS care for all HEI and HIV‐infected infants 14. Between 2014 and 2015, 15 HIV prevalence) operational districts (ODs) started the implementation of this IACM approach. A few studies have documented PMTCT in Cambodia, namely assessed the implementation of the PMTCT/Linked Response approach at its scaling 9, 10, the uptake of PMTCT interventions in some ODs 15 and provided a broader perspective of HIV elimination 16. However, to date none has evaluated PMTCT in ODs implementing the IACM approach.

Monitoring and evaluation as well as operational research are crucial to adapting and improving PMTCT programmes 17, 18, 19, 20, 21, 22, 23, 24. Moreover, cohort data collection, even on a subset of sites, has been recommended in order to get more reliable information and support in national data modelling systems 21. Since the introduction of option B+ a few studies have documented the PMTCT cascade indicators, and interventions contributing to improve retention and adherence to PMTCT programmes in sub‐Saharan Africa and in Asia 25, 26, 27, 28, 29, 30, 31, 32, 33, 34. The present study aims to analyse key indicators of the PMTCT service cascade following the implementation of the IACM approach in the 15 ODs in order to inform the Ministry of Health and adapt PMTCT interventions towards achieving the EMTCT targets.

## 2 Methods

### 2.1 Study Design, setting and sites

This study is an analysis of retrospective PMTCT cohort (or longitudinal) data, collected in 15 ODs through IACM monitoring tools, between 1 January 2014 and 31 December 2016. We also compared these PMTCT data with available (cross‐sectional) PMTCT data from the same ODs for the previous 2011 to 2013 period before the implementation of IACM.

#### 2.1.1 Study sites

By June 2016, all 15 IACM implementing ODs from nine provinces under the financial and technical support of different development partners including US‐CDC, USAID (under Flagship programme), UNICEF, AHF, CRS and ITM were included in the study. (The list of the 15 ODs is provided in Table 1; their populations and number of health centres is provided as additional file – Table S1).

**Table 1:** Number of HIV‐infected pregnant women and HEI receiving PMTCT and EID service in 15 ODs, after implementation of IACM, January 2014 to December 2016 (cohort data)

Operational Districts (ODs)	Identified HIV‐positive PW	HIV‐positive PW received ART (%)	HEI born and alive	HEI received ARV (%)	HEI aged ≥6 weeks	HEI received Cotrim (%)	HEI received DNA‐PCR test (%)	DNA‐PCR result+ (%)
1‐Chaktomuk	28	22 (78.6)	5	5 (100)	5	2 (40.0)	0 (0.0)	0
2‐Sen Sok	10	1 (10.0)	0	0 (0.0)	0	0 (0.0)	0 (0.0)	0
3‐Pursenchey	13	13 (100)	5	3 (60.0)	5	0 (0.0)	0 (0.0)	0
4‐Basac	26	20 (76.9)	0	0 (0.0)	0	0 (0.0)	0 (0.0)	0
5‐Mekong	1	1	0	0 (0.0)	0	0 (0.0)	0 (0.0)	0
6‐Battambang and Sangke	184	170 (92.4)	133	110 (82.7)	124	97 (78.2)	89 (71.8)	2
7‐Sampov Loun	63	56 (88.9)	40	36 (90.0)	36	34 (94.4)	31 (86.1)	0
8‐Siem Reap	124	121 (97.6)	83	71 (85.5)	78	49 (62.8)	57 (73.1)	0
9‐Mongkul Borey	79	77 (97.5)	50	34 (68.0)	50	34 (68.0)	40 (80.0)	2
10‐Poi Pet	88	87 (98.9)	30	27 (90.0)	28	14 (50.0)	21 (75.0)	0
11‐Pailin	41	40 (97.6)	20	20 (100)	19	18 (94.7)	17 (89.5)	0
12‐Sampov Meas	90	74 (82.2)	52	49 (94.2)	52	40 (76.9)	38 (73.1)	1
13‐Samrong	2	2 (100)	0	0 (0.0)	0	0 (0.0)	0 (0.0)	0
14‐Kampong Cham	145	141 (97.2)	100	86 (86.0)	98	88 (89.8)	92 (93.9)	2
15‐Sihanouk Ville	44	42 (95.5)	15	15 (100)	14	14 (100)	11 (73.3)	0
Total	938	867 (92.4)^a^	533	456 (85.6)	509	390 (73.2)^b^	396 (74.3)^b^	7 (1.8)

Cotrim, cotrimoxazole; HEI, HIV exposed infants; PW, pregnant women.

^a^Including all PW who initiated ART (abortions and lost to follow‐up); ^b^calculated using HEI born and alive as denominator.

#### 2.1.2 Description of the IACM approach

The Integrated Active Case Management (IACM) approach was introduced in 2013 to improve the notification and follow‐up of new HIV‐infected cases including HIV‐infected pregnant women. The IACM approach requires that all HIV testing/screening services in the districts notify all HIV cases detected to a case management coordinator (CMC) or their assistant (CMA) who ensure that all HIV detected cases are not lost and have access to the HIV service cascade. In each OD implementing the IACM approach, a CMC and a CMA were appointed to coordinate with relevant partners across HIV and maternal and child health (MCH) services to assist all HIV‐infected clients, including HIV‐infected pregnant women in accessing relevant HIV and MCH services 14. Similarly, for each confirmed HIV‐infected pregnant woman, the CMC and CMA also ensured that their HIV‐exposed infants (HEI) received ARV and cotrimoxazole prophylaxis as well as early infant diagnosis (EID) using DNA‐PCR tests at birth at the delivery facility, and were enrolled in paediatric AIDS care services for further EID tests if needed.

In addition, coordination mechanisms were set up to support the implementation of IACM approach. From OD level, new HIV‐infected cases were weekly communicated by the CMA to central HIV programme level. OD Sub‐Technical Working Groups (Sub‐TWG) on Active Case Management were also formed and met monthly to support and oversee the progresses and challenges in implementing IACM for key population, pregnant women and other general population. The CMC and CMA served as secretariat of this Sub‐TWG. At provincial and national levels, the implementation of IACM was coordinated and guided by Technical Working Groups (TWG). This TWG met every six months 14. In addition, a core group at the national HIV programme (NCHADS) met weekly to review the IACM data submitted from ODs and to provide feedbacks to CMC and CMA for follow‐up actions. NCHADS and relevant partners also met monthly to further review the IACM data to provide guidance and support to the provincial and OD levels to improve the implementation of IACM.

### 2.2 Study population

All HIV‐infected pregnant women, including those who were newly tested HIV positive and those already on ART who became pregnant, as well as their HIV‐exposed infants followed up between 1 January 2014 and 21 December 2016 in 15 ODs implementing the IACM approach were included in the analysis. All HIV‐infected pregnant women and their HIV‐exposed infants reported in the PMTCT/Linked Response programme in the same 15 ODs between the previous 2011 to 2013 period before IACM implementation were included using cross‐sectional (or aggregated data) for comparison in the analysis.

### 2.3 Data collection and analysis

A database was developed to enter all data from the IACM tools, which had been used since 2014. The CMA in each OD transferred individual data on tracking of identified HIV‐infected clients, including HIV‐infected pregnant women and their HEI, from the IACM tools in to the database under the support of IACM focal points and Data Management Unit of NCHADS (DMU/NCHADS). The CMA was required to send updated electronic database to DMU/NCHADS at central level every week.

Data were extracted from the IACM database and the following main indicators were calculated: (i) Percentage of HIV‐infected pregnant women who initiated ART among the total number of HIV‐infected pregnant women *identified*; (ii) Percentage of HEI who received ARV prophylaxis at birth for six weeks among all HEI born and alive (excluding stillbirths and neonatal deaths); (iii) Percentage of HEI aged ≥6 weeks who received at least 1 DNA PCR test among all HEI aged ≥6 weeks; (iv) Percentage of HEI aged ≥6 weeks who received cotrimoxazole prophylaxis among all HEI aged ≥6 weeks. These cohort data were compiled for the 15 ODs per year and a trend analysis was performed (Poisson regression with the year as covariate). They were then compared with PMTCT cross‐sectional routine programme data (as cohort data were not available for that period) from January 2011 to December 2013 before IACM implementation in the same 15 ODs, using Chi‐squared test. For consistency along the paper we used “ARV uptake” among HIV‐infected pregnant women when presenting data from 2011 to 2013 or when we compare the two time periods since some more simple ARV regimens were used up till 2013. However, only ART was used during the 2014 to 2016 period.

### 2.4 Ethical issues

Ethical approval for this study was obtained from the National Ethics Committee for Health Research (NECHR) in Cambodia and the Institutional Review Board (IRB) of the ITM and the Ethical Committee of Antwerp University (UZA) in Belgium. As this study consisted of routine data collection which were anonymized and kept confidential, no consent procedure was required.

## 3 Results

**Figure 1:**
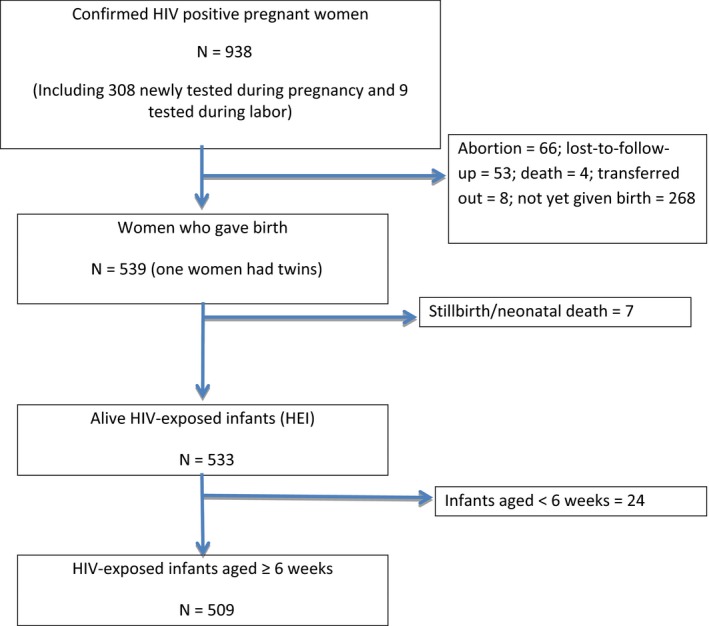
HIV‐infected pregnant women and HIV exposed infants born to HIV‐infected mothers between January 2014 and December 2016.

From a total of 938 HIV‐infected pregnant women identified through the IACM approach in the 15 IACM implementing ODs, 308 (32.8%) tested HIV positive during their pregnancy, 9 (1.0%) tested positive during labour, and 621 (66.2%) were HIV‐infected women already on ART became pregnant (Figure 1). Their median age was 32 years (range of 15 to 50 years). Among the 938 HIV‐infected pregnant women in the cohort, 66 (7.0%) opted for an abortion and 53 (5.7%) were reported as lost to follow‐up. Of the 66 women who opted for abortion, 26 (39.4%) were women newly tested HIV positive during pregnancy and 40 (60.6%) were known HIV‐infected women who became pregnant. Among the 53 women lost to follow‐up, 27 (50.9%) were women newly tested HIV positive and 26 (49.1%) were known HIV‐infected women who became pregnant. By 31 December 2016, 539 women in the cohort had given birth to 540 infants (one woman had twins) with only 533 infants alive, due to seven stillbirths/neonatal deaths. Among these 533 infants, 509 survived to age six weeks by the end of study follow‐up (Figure 1).

In regard to access to PMTCT services, our results show that among the 938 HIV‐infected pregnant women identified during the study period, 867 (92.4%) received ART during pregnancy and labour (Table 1). Subsequently, 456 (85.6%) of the 533 HEI alive during the study period received the 6‐week ARV prophylactic treatment according to the PMTCT guidelines (5), and 390 (73.2%) and 396 (74.3%) of them received cotrimoxazole and HIV‐DNA PCR test respectively. Among those reaching six weeks of age (n = 509), 390 (76.6%) and 396 (77.8%) received cotrimoxazole prophylaxis and HIV‐DNA PCR test, respectively. Among the 396 infants who received HIV‐DNA PCR test, 7 (1.8%) of them were found HIV positive (Table 1). PMTCT indicators varied per OD and per year between 2014 and 2016 (Tables 1 and 2).

**Table 2:** Total number and percentages of HIV‐positive pregnant women and HEI receiving PMTCT and EID service in 15 ODs between 2014 and 2016, by year (cohort data)

Year	HIV‐positive PW	HIV‐positive PW received ART or ARV proph (%)	HEI born and alive	HEI received ARV (%)	HEI received Cotrim (%)^a^	HEI received DNA‐PCR test (%)^a^	DNA‐PCR result+ (%)
2014	306	271 (88.6)	221	173 (82.0)	161 (72.9)	134 (60.6)	2 (1.5)
2015	323	305 (94.4)	190	171 (90.0)	155 (81.6)	160 (84.2)	3 (1.9)
2016	309	291 (94.2)	132	116 (87.9)	74 (56.1)	102 (77.3)	2 (2.0)

ARV proph, antiretroviral prophylaxis; Cotrim, cotrimoxazole; PW, pregnant women.

a Calculated using HEI born and alive as denominator.

All indicators showed an increase in 2014 to 2015; however, while the uptake of ART among HIV‐infected pregnant women remained stable, HEI indicators, in particular the uptake of cotrimoxazole by HEI, showed a decrease in 2016 (Table 2). No (log) linear trend was identified over the three‐year period 2014 to 2016 for the indicators (data not shown).

### 3.1 Comparison with PMTCT/linked response (cross‐sectional) routine data before the implementation of IACM during the previous 2011 to 2013 period

Aggregated routine Linked Response indicators in the same 15 ODs showed that 1273 HIV‐infected pregnant women and that 995 HEI were identified between 2011 and 2013 (Table 3). Of the 1273 HIV‐infected pregnant women identified, 554 (43.5%) were tested HIV positive during pregnancy, 26 (2%) during labour, and 693 (54.4%) were known HIV‐infected women who became pregnant. The comparison between IACM cohort data with these available data before IACM implementation (2011 to 2013), in the same 15 ODs showed that ARV uptake among identified HIV‐infected pregnant women, cotrimoxazole uptake and HIV‐DNA PCR testing coverage among HIV‐exposed infants increased significantly to reach over 90%, 73% and 74% during the second period 2014 to 2016 respectively (Table 3). However, ARV uptake among HEI decreased slightly, to reach 85% (*p *<* *0.001). The number of infants tested positive with PCR at six to eight weeks during IACM period were slightly lower than before IACM (n = 7 vs. n = 10 respectively) to reach an overall MTCT rate of 1.8% but the difference was not found statistically significant (Table 3).

**Table 3:** Comparison of the number of HIV‐positive pregnant women and HEI receiving PMTCT and EID services in 15 ODs before and after implementation of IACM

	Identified HIV ‐positive PW	HIV‐positive PW receiving ART/ARV proph (%)	HEI born	HEI received ARV (%)	HEI received Cotrim (%)	HEI received DNA‐PCR test (%)	DNA‐PCR positive result (%)
Before IACM Approach 2011 to 2013	1273	921 (72.3)	995	952 (95.7)	414 (41.6)^a^	410 (41.2)^a^	10 (2.4)
After IACM Approach 2014 to 2016	938	867 (92.4)	533	456 (85.6)	390 (73.2)^a^	396 (74.3)^a^	7 (1.8)
*p*‐value^b^	‐	*p *<* *0001	‐	*p *<* *0001	*p *<* *0001	*p *<* *0001	*p *=* *0.5073

ART, antiretroviral therapy; ARV proph, antiretroviral prophylaxis; HEI, HIV exposed infants; PW, pregnant women.

^a^Calculated using HEI born as denominator (as only data available for both period before and after IACM approach); ^b^chi square test was used.

## 4 Discussion

This PMTCT cascade study using cohort data showed that following the introduction of an IACM approach in 15 high risk (or HIV prevalence) ODs in Cambodia, an ART uptake of > 90% was achieved between 2014 and 2016 among identified HIV‐infected pregnant women. However, despite improved follow‐up of HIV pregnant women and HEI, coverage for cotrimoxazole uptake, and HIV‐DNA PCR testing among HEI remained below 75%. Our data also showed that there were some important variations in indicator performance between the 15 ODs. Nevertheless, the number of infants infected by vertical transmission remained below 5% and appeared to decrease (although not significantly) compared to the previous 2011 to 2013 period.

The important and significant increase in ARV uptake (+20%) among identified HIV‐infected pregnant women during the period 2014 to 2016 compared with the preceding 2011 to 2013 period suggests that the IACM approach was effective for improving ARV uptake among HIV‐infected pregnant women. Indeed, the IACM approach ensured a better follow‐up and as a result higher ARV uptake. However, it must be acknowledged that any comparison between the two time periods needs to be made with caution due to two different types of data collection (cohort vs. cross‐sectional data). In addition, the potential positive effect of option B+ and IACM approach on PMTCT cascade indicators is difficult to disentangle. However, option B+, implemented early 2013, could have already positively affected PMTCT indicators during the 2011 to 2013 period.

Our findings showing a high (92%) ARV uptake among *identified* HIV‐infected pregnant women in the 15 ODs are quite encouraging, suggesting that scaling‐up IACM nationwide could help to address this challenge. However, we could not calculate the proportion of ARV uptake among the *estimated* number of HIV‐infected pregnant women as this value is not available per each OD, and only provincial sub‐national estimates are available in Cambodia. Indeed, overall national data based on UNAIDS estimates show 79% ARV uptake among *estimated* HIV‐infected pregnant women in Cambodia in 2016 7, still below the 95% ARV coverage requested for EMTCT but in line with 2016 data from the 23 priority countries (78%) 1, 3, 35.

Despite active follow‐up, our study showed that ARV uptake among identified HEI was at 86% and decreased by 10% from the previous period. This decline may be due to decreased funds for Home‐Based Care NGOs to arrange transport for HIV‐infected women to give birth in hospitals offering ARV prophylaxis for HEI, as in Cambodia only selected hospitals where ART service is located are allowed to provide ARV prophylaxis for HEI. Also, it is likely that potential errors and/or double counting that can be associated with cross sectional data collection, in particular at the time of delivery, may overestimate the uptake and account for such higher rate during the 2011 to 2013 period. IACM approach implementation strengthened HEI follow‐up and data collection during the period 2014 to 2016 which seems supported not only by a higher ARV uptake among HIV‐infected PW as mentioned above, but also cotrimoxazole uptake and HIV‐DNA PCR testing that are at a much higher level (over 75%) during the period 2014 to 2016 compared with the previous period. However, the programme should reflect on how to ensure an appropriate follow‐up and subsequently a better coverage of PMTCT indicators during the six to eight weeks post‐partum period. It should also be acknowledged that the main focus of IACM approach was not pregnant women and PMTCT, as CMA had to deal with all new HIV‐positive cases among which pregnant women represented only a small proportion. The “post‐partum” gap remains a major challenge for PMTCT programmes in Cambodia, similarly to many other settings 1, 3, 19, 22, 23, 28. Continuously declining external funds represent a challenge for further improvement in PMTCT cascade outcomes. Finally, the number and coverage of HIV‐DNA PCR test in the 15 ODs increased dramatically over six years covered by this study. Similarly, the percentage of HIV‐positive children at six weeks was at around 2% in 2016.

PMTCT cascade outcomes varied between the 15 IACM approach implementing ODs. Some ODs which remained low performers received more mentoring, supervision and support from the national programme (NCHADS). In addition, regarding ODs located in Phnom Penh area (the five‐first ODs in the list of Table 1), there was a need for closer collaboration with national hospitals, which function largely independently from national programmes in Cambodia, as most of HIV‐infected pregnant women in Phnom Penh give birth in such national hospitals where accurate PMTCT services should also be provided. However, no specific intervention was designed to address this issue of national hospitals during the study period.

The NCHADS was well informed of these outcomes and gaps as they were involved in all processes of the study. Therefore, this study contributes to the decision of the national programme in designing and initiating interventions in 2017 after the study period to boost the implementation of IACM in order to improve HIV case detection including among pregnant women and to increase the uptake of HIV services cascade including PMTCT services cascade. First, NCHADS made a request to national hospitals in Phnom Penh to nominate relevant hospital staff as a member of OD Sub‐TWG on Active Case Management and to regularly attend the OD Sub‐TWG meeting to strengthen the coordination and referral of clients among HIV services in the Phnom Penh ODs and the national hospital. Second, NCHADS has revised and simplified IACM database, and developed IACM dashboard based on key indicators along the HIV cascades including PMTCT cascades for OD levels to regularly monitor the progress of the cascades. Finally, NCHADS and partners introduced and scaled up a Payment for Result Project providing incentive to ODs and provinces implementing IACM in an attempt to further improve their performance based on HIV cascades. These interventions are currently being strengthened and scaled‐up.

### 4.1 Study limitations

This study has a number of limitations. First, it used programme data, routinely collected through a newly introduced IACM database. Despite strengthened supervision and monitoring and data managed by designated data managers, recording mistakes cannot be excluded. Second, and related to the first point, HEI data are missing for infants born beyond the study data collection period 31 December 2016 (Figure 1). An analysis of characteristics and ARV uptake of these infants’ mothers were similar and suggests similar HEI data as well (data not shown). However, within this programme data and cascade analysis we choose to be conservative and not to impute or extrapolate from the data. Third, we did not collect cohort data in control ODs where the IACM approach was not implemented. The comparison between cohort data and cross‐sectional data from the period before IACM is not ideal, but they were the only data available and provided some indications of trends over time using the IACM approach. In addition, except for early HIV‐DNA PCR testing among HEI, no data/indicators were collected in the post‐partum period regarding the mode and duration of infant feeding and possible infant testing and MTCT rate at the end of breastfeeding period. Finally, qualitative data that could have helped understanding the reasons for the observed gaps in the PMTCT cascade were not collected.

## 5 Conclusions

Our findings show that PMTCT indicators in particular ARV uptake among HIV‐infected pregnant women, and HIV‐DNA PCR testing among HEI showed a substantial increase in 2014 and 2015 compared with the previous period. The implementation of option B+ and IACM may have contributed to the improvement of PMTCT cascade. However, some gaps in accessing PMTCT services along the HIV cascade persist and need to be addressed**.**

## Competing interests

We certify that there is no competing interests for all authors involved in this study.

## Authors’ contributions

SS, PSL, VO and TD conceived and designed the study. SS, VK, VO and BN coordinated/implemented data collection. SM, SS, VK and AK conducted data management. SS, RT, AK and TD analysed data. SS, VK, CVM, VO, LF and TD wrote the manuscript. All authors provided input in manuscript writing and approved the final manuscript.

## Abbreviations

ANC: Antenatal Care
ART: Antiretroviral Therapy
ARV: Antiretroviral
CD4: Cluster of Differentiation 4
CMA: Case Management Assistant
CMC: Case Management Coordinator
DNA‐PCR: Deoxyribonucleic Acid‐Polymerase Chain Reaction
EMTCT: Elimination of Mother‐to‐ Child Transmission of HIV
HEI: HIV Exposed Infant
IACM: Integrated Active Case Management
OD: Operational District
PMTCT: Prevention of Mother‐to‐ Child Transmission of HIV
PW: Pregnant women
TWG: Technical Working Group
UNAIDS: United Nations Programs on HIV and AIDS
WHO: World Health Organization

## Supporting information


**Table S1.** List of the 15 operational districts included in the study, provinces, IACM implementing dates and partners, populations and number of health centers (2016).
