# Supplementary material for: Access to prevention of mother‐to‐child transmission of HIV along HIV services cascade through integrated active case management in 15 operational districts in Cambodia
Source: J Int AIDS Soc. 2019 Oct 20;22(10):e25388. doi: 10.1002/jia2.25388 (PMC6801228; doi:10.1002/jia2.25388)
Supplement: Supplementary file 1 — Table S1. List of the 15 operational districts included in the study, provinces, IACM implementing dates and partners, populations and number of health centers (2016). [file JIA2-22-e25388-s001.docx]

Additional file

Table S1: ***List of the 15 operational districts included in the study, provinces, IACM implementing dates and partners, populations and number of health centers (2016)***

| **Operational Districts (ODs)** | | **City/**  **Provinces** | **Supporting Partners** | **IACM Starting Date** | **Population in 2016** | **Number of health centers** |
| --- | --- | --- | --- | --- | --- | --- |
|  |  |  |  |  |  |  |
| 1-Chaktomuk | | Phnom Penh | USAID/Flagship | September, 2014 | 421,240 | 5 |
| 2-Sen Sok | | Phnom Penh | USAID/Flagship | September, 2014 | 137,042 | 5 |
| 3-Pursenchey | | Phnom Penh | USAID/Flagship | September, 2014 | 221,917 | 6 |
| 4-Basac | | Phnom Penh | USAID/Flagship | September, 2014 | 322,723 | 7 |
| 5-Mekong | | Phnom Penh | UNICEF | May, 2015 | 219,787 | 4 |
| 6-Battambang and Sangke | | Battambang | US-CDC | March, 2014 | 576,986 | 38 |
| 7-Sampov Loun | | Battambang | US-CDC | April, 2014 | 162,471 | 10 |
| 8-Siem Reap | | Siem Reap | USAID/Flagship | September, 2014 | 385,976 | 29 |
| 9-Mongkul Borey | | Banteay Meanchey | US-CDC | April, 14 | 247,530 | 23 |
| 10-Poi Pet | | Banteay Meanchey | US-CDC | April, 2014 | 206,423 | 16 |
| 11-Pailin | | Pailin | CRS | May, 2014 | 112,509 | 6 |
| 12-Sampov Meas | | Pursat | US-CDC | April, 2014 | 429,391 | 12 |
| 13-Samrong | | Odor Meanchey | ITM | September, 2015 | 142,545 | 23 |
| 14-Kampong Cham | | Kampong Cham | USAID/Flagship | June, 2014 | 168,873 | 14 |
| 15-Sihanouk Ville | | Sihanouk Ville | AHF | February, 2014 | 203,844 | 14 |
| **Total** | | **15 ODs** |  |  | **3,959,257** | **198** |
|  |  |  |  |  |  |  |
